# Deep-learning recognition and tracking of individual nanotubes in low-contrast microscopy videos

**DOI:** 10.3762/bjnano.16.96

**Published:** 2025-08-13

**Authors:** Vladimir Pimonov, Said Tahir, Vincent Jourdain

**Affiliations:** 1 Laboratoire Charles Coulomb, Univ Montpellier, CNRS, Montpellier, France

**Keywords:** carbon nanotubes growth kinetics, deep learning, in situ microscopy, object recognition and tracking, polarization microscopy

## Abstract

This study addresses the challenge of analyzing the growth kinetics of carbon nanotubes using in situ homodyne polarization microscopy (HPM) by developing an automated deep learning (DL) approach. A Mask-RCNN architecture, enhanced with a ResNet-FPN backbone, was employed to recognize and track individual nanotubes in microscopy videos, significantly improving the efficiency and reproducibility of kinetic data extraction. The method involves a series of video processing steps to enhance contrast and used differential treatment techniques to manage low signal and fast kinetics. The DL model demonstrates consistency with manual measurements and increased throughput, laying the foundation for statistical studies of nanotube growth. The approach can be adapted for other types of in situ microscopy studies, emphasizing the importance of automation in high-throughput data acquisition for research on individual nano-objects.

## Introduction

Carbon nanotubes (CNTs), discovered over three decades ago, continue to present unresolved questions and challenges. Their exceptional properties, both theoretically [1–2] and experimentally demonstrated [3], make them desirable for electronic and optical devices. However, the widespread application of CNTs is hindered by the lack of control over their structure during growth. Therefore, developing highly selective synthesis methods is crucial for advancing CNT-based devices. This requires a deep understanding of the relationship between nanotube structure and selectivity, particularly kinetic selectivity. To address this, we developed a method based on in situ homodyne polarization microscopy (HPM), which is highly sensitive and can detect changes in optical absorption caused by a single carbon nanotube. The technique allows for imaging tens to hundreds of individual carbon nanotubes during growth at up to 40 frames per second [4]. However, the vast amount of information generated requires meticulous and time-consuming analysis to extract kinetic data.

This challenge, common in imaging-related fields, can be addressed through advances in artificial intelligence (AI), particularly in computer vision (CV). Early attempts to automate visual information processing began over three decades ago with one of the first convolutional neural networks recognizing handwritten zip codes on postage envelopes [5]. Since then, CV algorithms have significantly progressed, finding numerous applications in scientific research. Deep learning models have been used to identify two-dimensional materials in microscopic images [6], characterize mineral composition in scanning electron microscopy (SEM) samples [7], and determine nanotube chirality from transmission electron microscopy images [8]. Here, we report on using a deep learning algorithm to automatically recognize and track individual carbon nanotubes during growth, captured in polarization microscopy video sequences.

## Materials and Methods

### CNT growth

The experimental setup was previously described in [4,9]. In short, horizontally aligned carbon nanotubes (HA-CNTs) were synthesized inside a miniature chemical vapor deposition (CVD) cell with an optical window (Linkam TS1500). ST-cut quartz and iron nanoparticles served as substrate and catalyst, respectively. Ethanol and argon were, respectively, used as carbon precursor and carrier gas. Oxygen and water sensors monitored gas-phase contaminants at the outlet line.

### In situ microscopy

Nanotube growth was imaged in situ using a custom-built optical setup for homodyne polarization microscopy. A supercontinuum source (Fianium SC-400-4, 2 ps pulses, 40 MHz, spectral range 400–2000 nm) provided white light excitation across the visible spectrum. Two crossed polarizers were employed, with a polarizer and analyzer used to enhance the scattered field from the nanotubes relative to the stronger reflected field from the substrate. A low-pass optical filter with a cutoff wavelength of 700 nm was placed after the analyzer to filter out blackbody radiation generated by the heating crucible. A long-distance objective (Nikon Plan Fluor ELWD 20× 0.45 C L) was used for illumination and collection. Growth process videos were captured using a digital camera (Hamamatsu c11440 ORCA-Flash4.0 LT) with maximum acquisition rate up to 40 frames per second (fps).

### Video processing

To extract kinetic data from each nanotube, the raw videos were first processed to enhance contrast. The detailed processing methodology is provided in Supporting Information File 1. In short, after alignment the initial frame rate, typically between 40 and 25 fps, was reduced by averaging frames to one frame per second. This operation boosts further analysis and improves signal-to-noise ratio without compromising kinetic data quality, as the nanotube growth rate is typically of the order of 0.5 µm·s^−1^ and the localization precision of the optical setup is 0.33 µm. Frames were then aligned using a template matching algorithm from the OpenCV Python library. Shade correction was applied to compensate for uneven illumination caused by the optics. Residual noise was reduced using fast Fourier transform (FFT) band-pass filtering, and object edge contrast was enhanced using Gaussian difference filtering. Finally, image contrast was optimized through histogram equalization [10].

### Deep learning model

The deep learning model and its training are detailed in Supporting Information File 1. In short, an image recognition system was implemented using the PyTorch library [11]. For recognizing nanotubes in videos, we employed the Mask-RCNN architecture [12] with the ResNet-FPN neural network combination as a backbone [13–14], pre-trained on the Microsoft COCO-2017 dataset [15–16]. Training was conducted on graphics processing units provided by Google Colab [17] over 150 epochs with a learning rate of 0.005, decreasing by 10% every 5 epochs. A weight decay of 0.0005 with momentum of 0.9 was used.

## Results and Discussion

**Figure 1:**
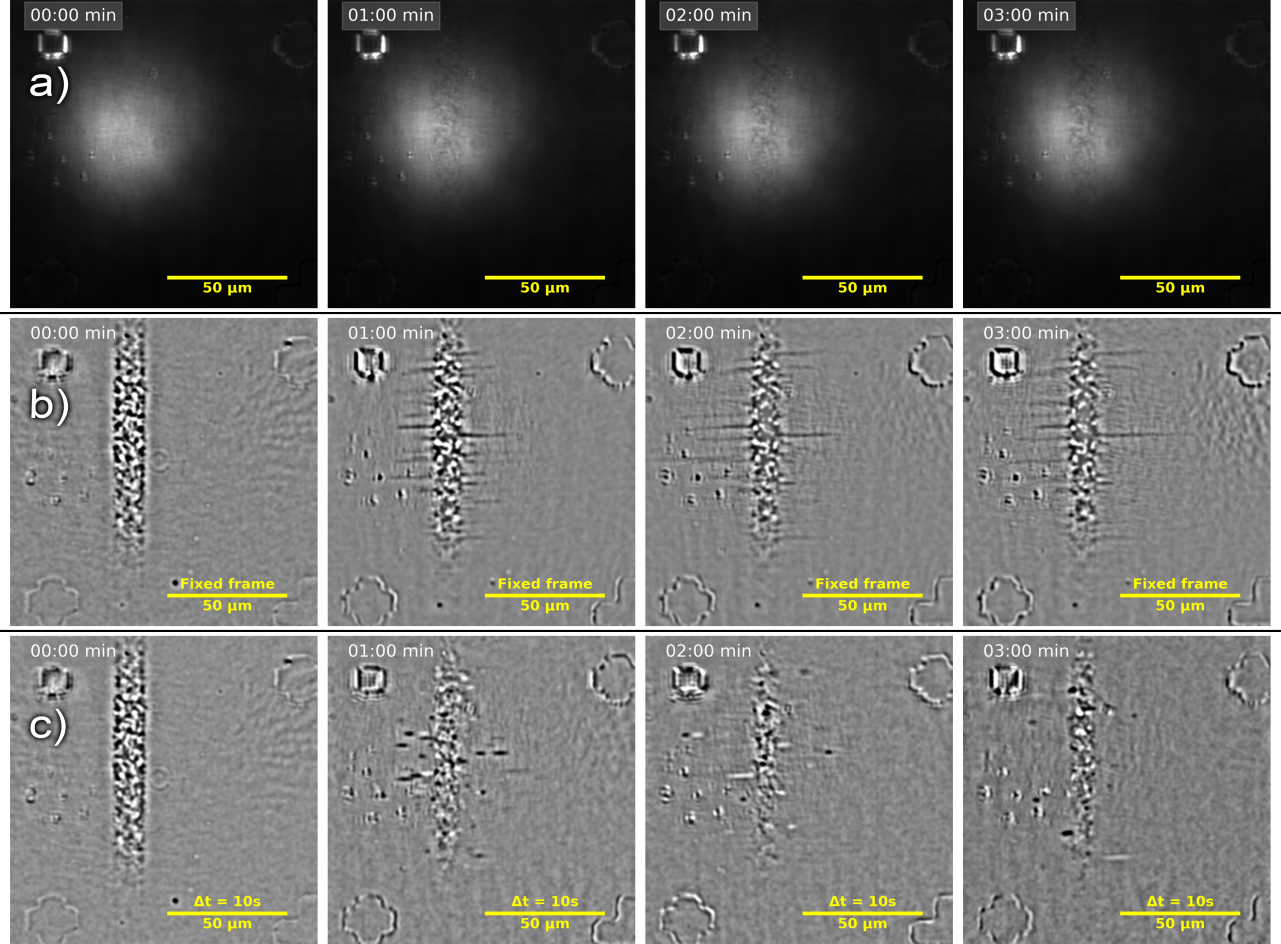
Snapshots from (a) the raw video of carbon nanotube (CNT) synthesis on a stable cut (ST) quartz substrate and from the fully processed video using (b) fixed-frame and (c) differential shading correction (with a 10 s delay). The thick vertical line in each image indicates the catalyst line. Optical markers (i.e., squares, crosses, and L-shapes) are visible in the corners. CNTs appear as thin horizontal lines in the fixed-frame images and as segmented features in the differential images. Grown CNTs show dark contrast in both treatments, while shrinking CNTs are only visible in the differential images and appear as bright segments.

In situ videos of CNT growth obtained using HPM contain extensive kinetic information. However, the low contrast of raw videos (Figure 1a) is inadequate for kinetic assessment, necessitating raw video processing for statistical data acquisition on nanotube growth kinetics. A rolling-frame method (also called differential-treatment) was developed to enhance contrast (see details in Supporting Information File 1). This approach, which is a variation of the shade correction commonly used in image processing, involves subtracting a background snapshot containing the illumination profile from the image [18–19]. Typically, the first frame (Figure 1a, left image) of a sequence is used as the background (Figure 1b). However, using the frames with a fixed delay time (chosen between 5 and 30 s, see Supporting Information File 1, Figure S2) significantly increases contrast, by up to an order of magnitude (Figure 1c as well as Supporting Information File 1, Figure S3, and Supporting Information Files 2 and 5). The method is termed “differential” or “rolling frame” shade correction due to the rolling of the background and processed frames.

In such differential videos, the length of a nanotube segment is proportional to its instantaneous rate, as described in Equation S5 (Supporting Information File 1), which adds useful information to the video. Additionally, differential videos capture other processes causing local changes in optical absorption. For instance, if the nanotube structure (also called helicity or chirality) changes during growth, this manifests as a second segment moving synchronously with the first one: The new chirality appears either as a bright segment if it has lower optical absorption, or as a dark segment otherwise. If the nanotube switches from growth to shrinkage, it appears as a single bright segment moving backward, corresponding to lower optical absorption [20].

**Figure 2:**
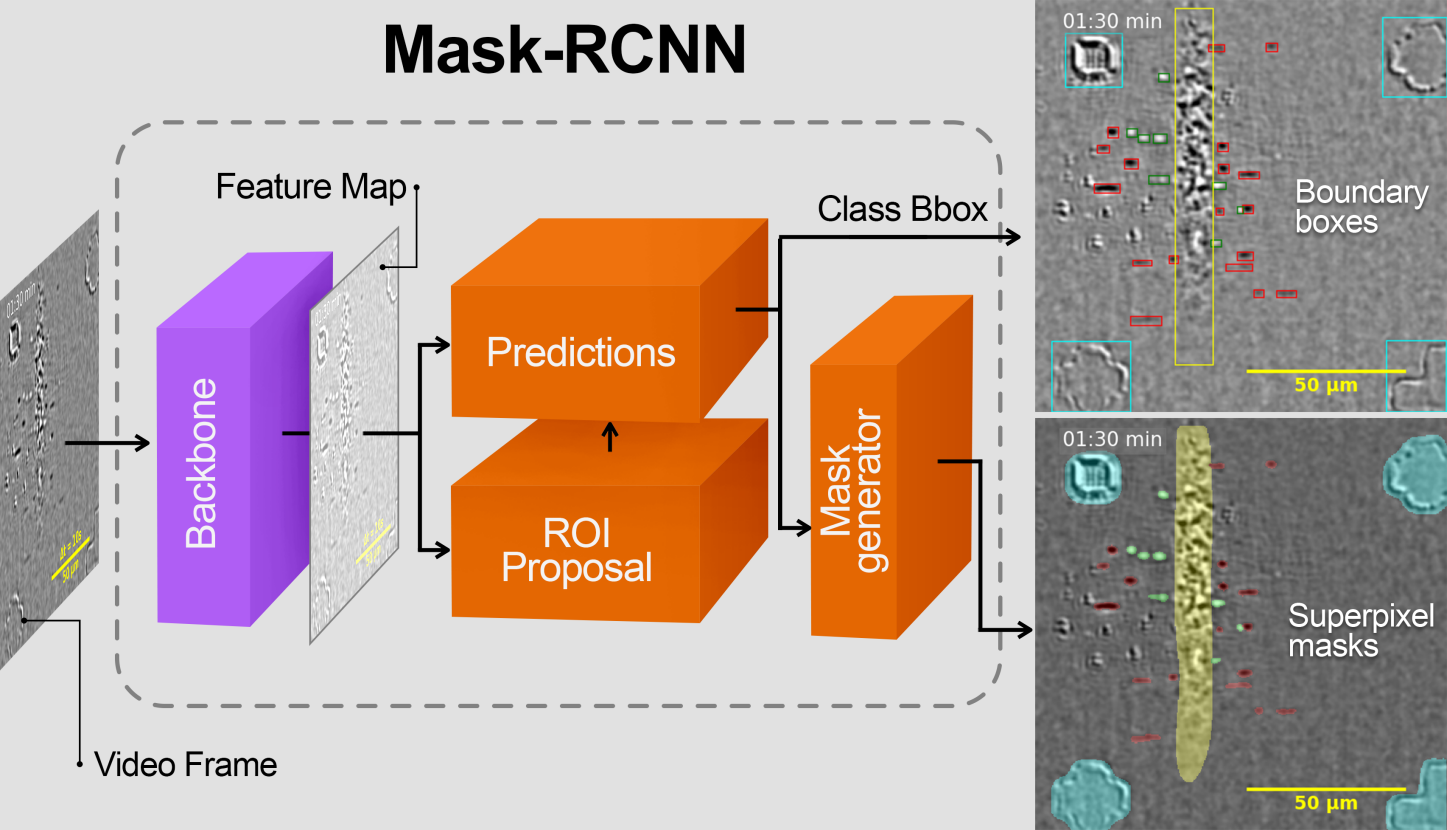
Schematic of object recognition system based on Mask-RCNN. Colors on the right insets highlight growing nanotube segments (red), structural changes (green), catalyst line (yellow), and optical marks (blue).

We developed a deep learning model to recognize and track both growing nanotubes (dark segments) and structural changes (bright segments) in such differential videos. The model was also trained at recognizing optical marks and catalyst lines (Figure 2) [20]. Kinetic data extraction proceeded in the following steps: (1) object recognition, (2) tracking of recognized objects, (3) verification of tracking, and (4) kinetic curve extraction and analysis. The initial stage of the process utilizes the Mask-RCNN neural network implemented in PyTorch Python library [11]. This architecture integrates several neural networks in a single pipeline (Figure 2). The image is first processed by the backbone network to produce a feature map. Region of interest (ROI) proposal and prediction networks then localize boundary boxes of the objects and classify them. Finally, a fully connected convolutional network generates a super pixel mask of the recognized objects [21].

The training dataset consisted of 580 manually labeled images from various videos. During training, the dataset was split into a training set of 550 images and a validation set of 30 images. In image segmentation, this data amount does not hinder model quality, and subsets as small as 30 images have been reported sufficient in some cases [22–23]. Nonetheless, we analyzed the dependence of the model prediction quality on the training set size (see Supporting Information File 1, Figure S6 and Table S1). Given the varying quality of our videos, augmentations were applied to account for possible variations in image brightness, contrast, and nanotube localization (Supporting Information File 1, Figure S4). This approach not only expanded the dataset without additional manual labeling but also increased the robustness and stability of the trained model [24] (Supporting Information File 1, Table S1).

The fully trained model detected segments corresponding to nanotube growth and structural changes, as well as optical marks and catalyst lines (Supporting Information File 1, Figure S5, and Supporting Information File 2). This process was conducted frame by frame through the video. To extract the growth kinetics of each nanotube, the as-recognized objects were tracked using the Hungarian method [25] and Kalman filter (or linear quadratic estimation) [26], which are widely used for object tracking [27]. The Hungarian algorithm matched masks across successive frames. However, some video frames are unrecognizable due to imaging artifacts, illumination instability, or uncompensated vibrations. Following the initial tracking stage, all segments were grouped into clusters of varying sizes, corresponding to objects recognized across consecutive frames. The clusters were then subjected to a Kalman filter to merge segments corresponding to the same nanotube (Supporting Information File 3).

**Figure 3:**
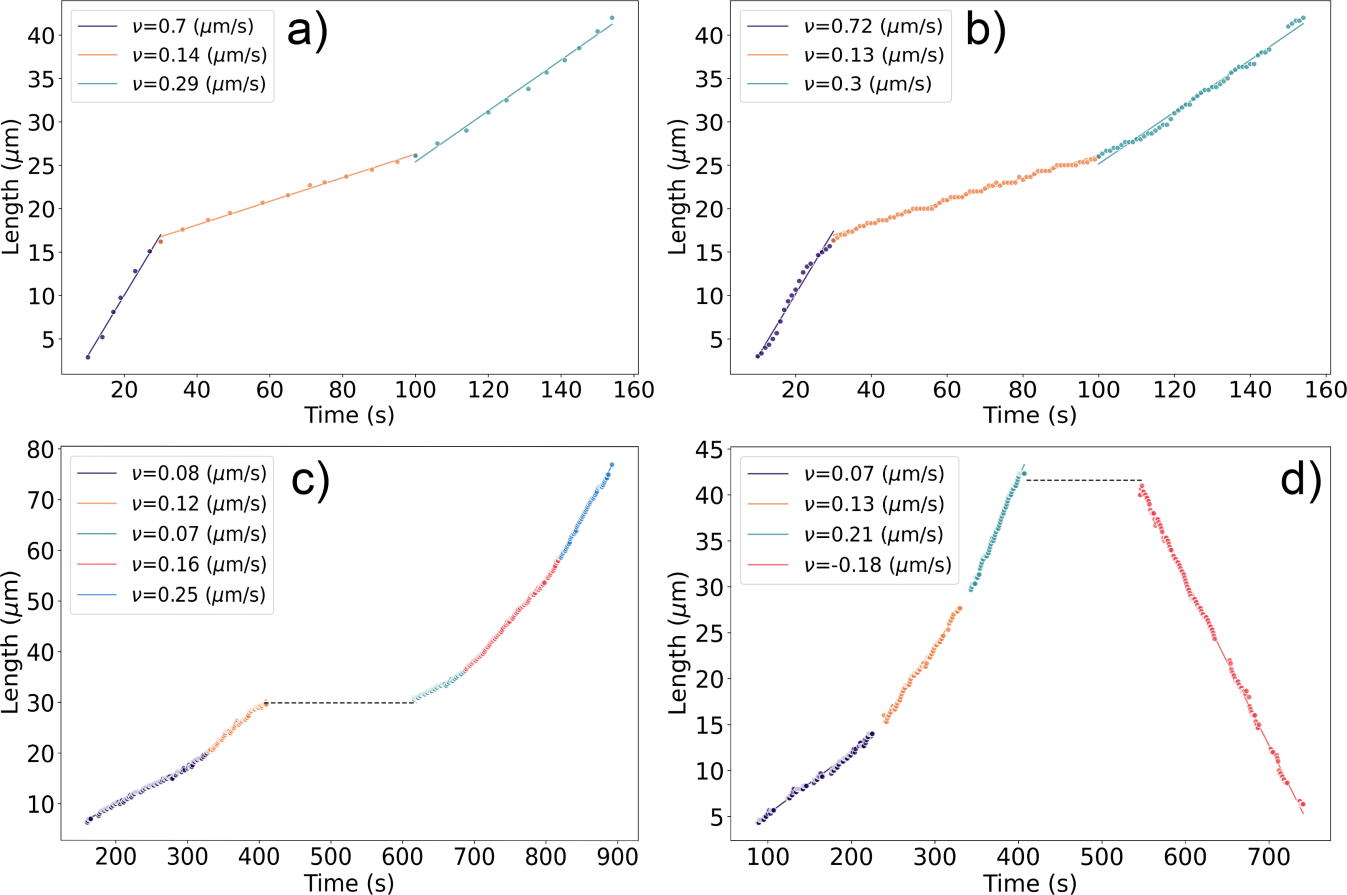
Kinetic growth curves of individual carbon nanotubes. Panels (a) and (b) show the same nanotube analyzed manually and with an automatic recognition system, respectively. Panels (c) and (d) depict nanotubes grown with a pause between two growth stages, and between growth and etching, respectively. Different colors indicate segments with distinct growth rates. Data points represent measured values, while solid lines show linear fits for each constant-growth segment (growth rates, indicated by slopes, are provided in the inset legend). Black dashed lines mark the time intervals where growth was paused.

The information about tracked segments is entered into tables for final manual verification and labeling of events. This manual step remains essential due to the complexity of nanotube kinetics, which involves switches between growth, pauses, shrinkage, and structure change during growth [20]. Pauses (Figure 3c,d) cannot be efficiently traced by the Hungarian method or Kalman filter, necessitating manual verification to ensure correct assignment of newly grown segments to the same or another close nanotube (Figure 3). Despite this final manual check, automating recognition and tracking steps has accelerated kinetic extraction, significantly enhanced the time resolution of kinetic curves (Figure 3b–d), and increased the repeatability of kinetic measurements. A comparison of manually measured and DL model-extracted results is shown in Figure 3a,b.

**Figure 4:**
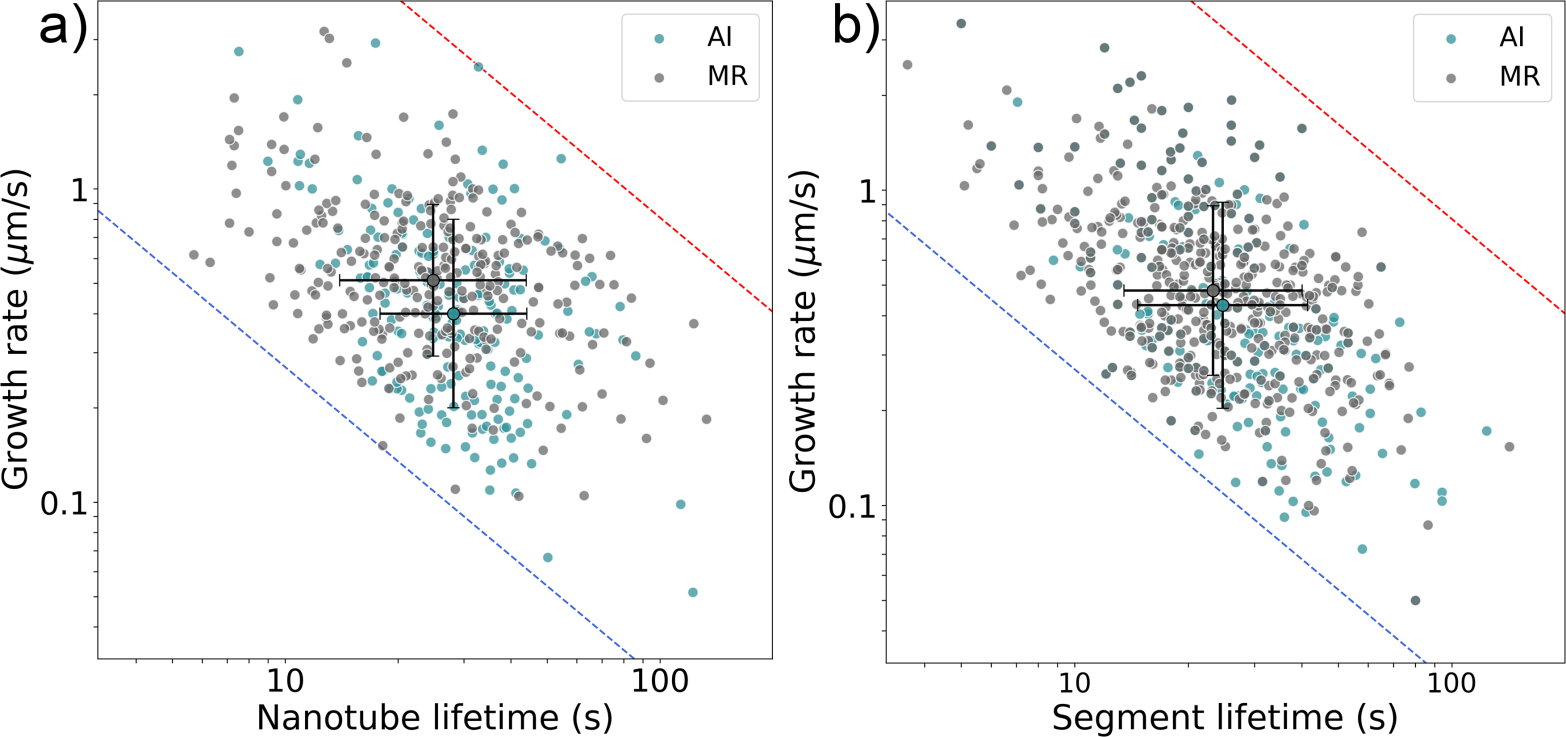
Plots of growth duration (lifetime) versus growth rate for individual carbon nanotubes. Panel (a) shows nanotubes with constant growth rates, while panel (b) includes nanotubes with varying growth rates. Green dots represent data obtained using the automated recognition system (AI), and grey dots correspond to manual measurements (MR). Large dots indicate the mean values of each distribution, with whiskers representing one standard deviation along each axis. Dashed lines mark nanotube lengths of constant value: blue for 2.7 µm (the shortest measured tube), and red for 81 µm (the maximum length observable within the field of view of the HPM imaging system).

Figure 4 compares the main kinetic parameters extracted manually and using the AI-assisted method from in situ videos of nanotube growth performed at the same growth temperature and ethanol partial pressure. In Figure 4a, the lifetime and segment growth duration represent the time during which the nanotube grew at a constant rate (see dots clusters of the same color in Figure 4). Linearly grown nanotubes exhibit constant growth rates from start to finish, observed in about half of the cases. The other half show one or more stochastic rate changes, or even growth–shrinkage switches separated by pauses [20]. In both cases, the close alignment of distribution centers, overlapping standard deviations, and similar overall profiles of the kinetic parameters extracted manually (MR) and via AI-assisted tracking confirm the consistency between the two approaches. Statistical testing (Table 1) supports this: While one instance of growth rate comparison (Figure 4a) yielded a statistically significant difference (*p* = 0.026), the associated effect size was small (*d* = 0.213), indicating only a minor practical difference. All other comparisons showed no statistically significant differences and negligible effect sizes, further underscoring the agreement between methods. This consistency validates the applicability of both manual and AI-assisted techniques for analyzing both linear and non-linear CNT growth kinetics. The difference in the number of data points shown in Figure 4 arises from the exclusion of videos manually analyzed during model training from the AI-based analysis, in order to avoid bias and ensure an independent performance assessment. The AI-extracted data in Figure 4 represents a small fraction of all data acquired using this method, forming the foundation for developing a statistically supported model of carbon nanotube growth kinetics. On average, extracting kinetic data from a single video takes approximately 6 h for a time resolution of 5 s using the manual method, compared to just 2 h for a time resolution of 1 s with the deep learning model, meaning that the throughput was increased by about a factor of 15 (Figure 3a,b).

**Table 1:** Statistical metrics corresponding to the data shown in Figure 4, comparing kinetic parameters derived from manual and AI-assisted analyses.

Plot ref.	Kinetic parameter	Mean ± std	p-value	Cohen’s *d*	Interpretation

Figure 4a	growth rate AI	0.51 ± 0.43	0.026	0.213	difference significant, but minor
	growth rate MR	0.59 ± 0.39			
	lifetime AI	31.2 ± 16.1	0.226	0.112	insignificant difference
	lifetime MR	29.2 ± 18.6			
Figure 4b	growth rate AI	0.55 ± 0.48	0.66	0.03	insignificant difference
	growth rate MR	0.57 ± 0.41			
	segment growth durations AI	27.4 ± 16.4	0.37	0.069	insignificant difference
	segment growth durations MR	26.3 ± 15.2			

## Conclusion

We have identified several critical steps in developing a DL model for extracting kinetic data from in situ microscopy videos of individual nano-objects. A specific challenge is the low signal from nano-objects and fast kinetics on the video time scale. Applying differential treatment not only drastically improves contrast but also enhances a human operator’s understanding of ongoing processes. This is crucial for manual recognition and tracking and for properly labeling the dataset used to train the DL model, allowing for comparison of data obtained by both methods to validate their coherence. Properly designed training datasets and augmentations improve model robustness and stability without increasing training data volume.

Despite significant advancements, there is room for improvement in processing algorithms. In particular, manual verification and labeling of tracked nanotubes, particularly for complex cases, remains essential and is currently the most time-consuming step in the process [20]. Currently, brightness and contrast adjustments rely on empirical hyperparameters, which could benefit from self-tuning or non-parametric algorithms. The same applies to FFT and Gaussian filtering parameters, which are presently constant. Additionally, the AI-based algorithm for kinetic extraction from differentially treated videos can be refined, regardless of the performance comparable to the current state-of-the-art models [28]. Expanding the dataset to include more diverse video quality and nanotube localization can lead to more stable models [29]. Moreover, replacing computationally demanding models like Mask-RCNN with smaller models could enhance overall performance, although potentially reducing recognition accuracy.

Automating recognition and tracking is essential for high-throughput video analysis, which is critical for understanding and modeling complex nanoscale phenomena. The differential video treatment approach, combined with the deep learning-enhanced kinetic extraction framework presented in this work, enabled the extraction of detailed kinetic parameters, specifically, growth rates, lifetimes, and final segment lengths of over 2000 individual carbon nanotubes observed across more than 50 in situ homodyne polarization microscopy videos acquired under a range of synthesis pressures and temperatures. This statistic is an order of magnitude larger than any other study on the growth kinetics of individual carbon nanotubes realized using other methods [30–31].

This large dataset provided unprecedented statistical insight into CNT growth kinetics, revealing complex behaviors such as intermittent switching between growth, pause, and etching modes, even under nominally constant synthesis conditions [20]. These observations, further supported by complementary Raman spectroscopy, served as a foundation for the development of a new mechanistic model of CNT growth, in which the structure and dynamics of the CNT edge at the catalyst interface govern transitions between kinetic regimes [32].

The versatility of the proposed method extends beyond the specific imaging modality used in this study. Its robustness makes it applicable to other in situ imaging platforms for nanomaterials, such as environmental transmission electron microscopy (ETEM) of CNTs [31,33] and environmental scanning electron microscopy (ESEM) of graphene growth and etching processes [34].

## Supporting Information

Supporting Information includes a PDF file with the expanded description of data processing and ten videos derived from two distinct experimental samples. The videos (Supporting Information Files 2–11) illustrate various stages of video processing, object recognition, and nanotube tracking as described below. **Note**: Recognition and tracking were performed only on the video in Supporting Information File 4 as it was excluded from model training to avoid bias. The image sequences in Supporting Information Files 6–11 can be viewed using the open source free software ImageJ [35–36].

File 1 Additional information regarding video processing. Expanded description of the differential video processing, comprehensive explanation of model training process, evaluation of different models, and description of tracking process.

File 2 This video shows raw, fixed-frame, and rolling-frame (differentially processed) in situ sequences.

File 3 This video presents the differentially processed sequence after object recognition. Masks and bounding boxes are superimposed on the left and right halves of the frame, respectively.

File 4 This finalized differential video demonstrates the complete recognition and tracking pipeline, including application of the Hungarian algorithm, Kalman filtering, and manual verification. Masks are displayed on the left side and bounding boxes on the right.

File 5 This video shows raw, fixed-frame, and rolling-frame (differentially processed) in situ sequences.

File 6 This video shows an uncompressed .tiff image sequence of the raw sequence corresponding to the content shown in the video in Supporting Information File 4.

File 7 This video shows an uncompressed .tiff image sequence of the fixed-frame processed sequences corresponding to the content shown in the video in Supporting Information File 4.

File 8 This video shows an uncompressed .tiff image sequence of the rolling-frame (differentially processed) sequences corresponding to the content shown in the video in Supporting Information File 4.

File 9 This video shows an uncompressed .tiff image sequence of the raw sequence corresponding to the content shown in the video in Supporting Information File 5.

File 10 This video shows an uncompressed .tiff image sequence of the fixed-frame processed sequences corresponding to the content shown in in the video Supporting Information File 5.

File 11 This video shows an uncompressed .tiff image sequence of the rolling-frame (differentially processed) sequences corresponding to the content shown in the video in Supporting Information File 5.

## Data Availability

The code used for training the model, with the example of model and training data is available as a zenodo repository "Training Script for Mask R-CNN on In-Situ Homodyne Microscopy Videos of Carbon Nanotube Growth" https://doi.org/10.5281/zenodo.15697833.
