# Supplementary material for: Deep-learning recognition and tracking of individual nanotubes in low-contrast microscopy videos
Source: Beilstein J Nanotechnol. 2025 Aug 13;16:1316–24. doi: 10.3762/bjnano.16.96 (PMC12362302; doi:10.3762/bjnano.16.96)
Supplement: File 1 — Additional information regarding video processing. Expanded description of the differential video processing, comprehensive explanation of model training process, evaluation of different models, and description of tracking process. [file Beilstein_J_Nanotechnol-16-1316-s001.pdf]

BEILSTEIN JOURNAL OF NANOTECHNOLOGY

Supporting Information

for

Deep-learning recognition and tracking of individual nanotubes in low-contrast microscopy videos

Vladimir Pimonov, Said Tahir and Vincent Jourdain

Beilstein J. Nanotechnol. **2025,** *16,* 1316–1324. doi:10.3762/bjnano.16.96

Additional information regarding video processing. Expanded description of the differential video processing, comprehensive explanation of model training process, evaluation of different models, and description of tracking process

License and Terms: This is a supporting information file under the terms of the Creative Commons Attribution License (https://creativecommons.org/licenses/by/4.0). Please note that the reuse, redistribution and reproduction in particular requires that the author(s) and source are credited and that individual graphics may be subject to special legal provisions.
The license is subject to the *Beilstein Journal of Nanotechnology* terms and conditions: (https://www.beilstein-journals.org/bjnano/terms)

Image processing and contrast enhancement

The in situ homodyne microscopy setup allows for capturing the growth of carbon nanotubes under real growth conditions with a frame rate of up to 45 frames per second (fps). The effective field of view is approximately 80 μm, limited by the width of the nearly parallel light beam at the sample's surface. Figure S1a shows several frames from a typical raw in situ video of nanotube growth. The nanotube contrast C is defined as:

$$\mathrm{C} = \frac{I - I_0}{I_0} \qquad \text{(S1)}$$

where I_0 is the mean background value and I is the mean nanotube signal value. It is apparent from raw videos (Figure S1a) that the nanotube contrast is usually too low for growth behavior analysis. Therefore, the image sequences must be processed beforehand. Figure S1b shows snapshots from the in situ video after standard treatment procedures consisting of the following steps:

1. frame alignment and framerate reduction,
2. illumination correction,
3. edge enhancement.

The first step is indispensable for further analysis, whether manual or automatic. Discrepancies between frames during the video may be caused by factors such as equipment vibrations, sample thermal expansion, and displacement of the microscope table. To ensure the spatial stability of the filmed objects, we combined two complementary methods. To eliminate large displacements (more than 1 μm or ~3 pixels), we applied the template matching algorithm from the OpenCV library [1]. Then, to compensate for smaller discrepancies, we reduced the number of frames by averaging them over 1 s. This resulted in videos with a frame rate of 1 fps. Averaging

might not be suitable for videos capturing rapid events; but in our experiments, the growth rates of our nanotubes vary within the range of 0.5–1.5 µm/s, and the localization precision of the imaging setup is around 0.3 µm/pix. Hence, frame averaging did not corrupt the kinetic information of carbon nanotube growth.

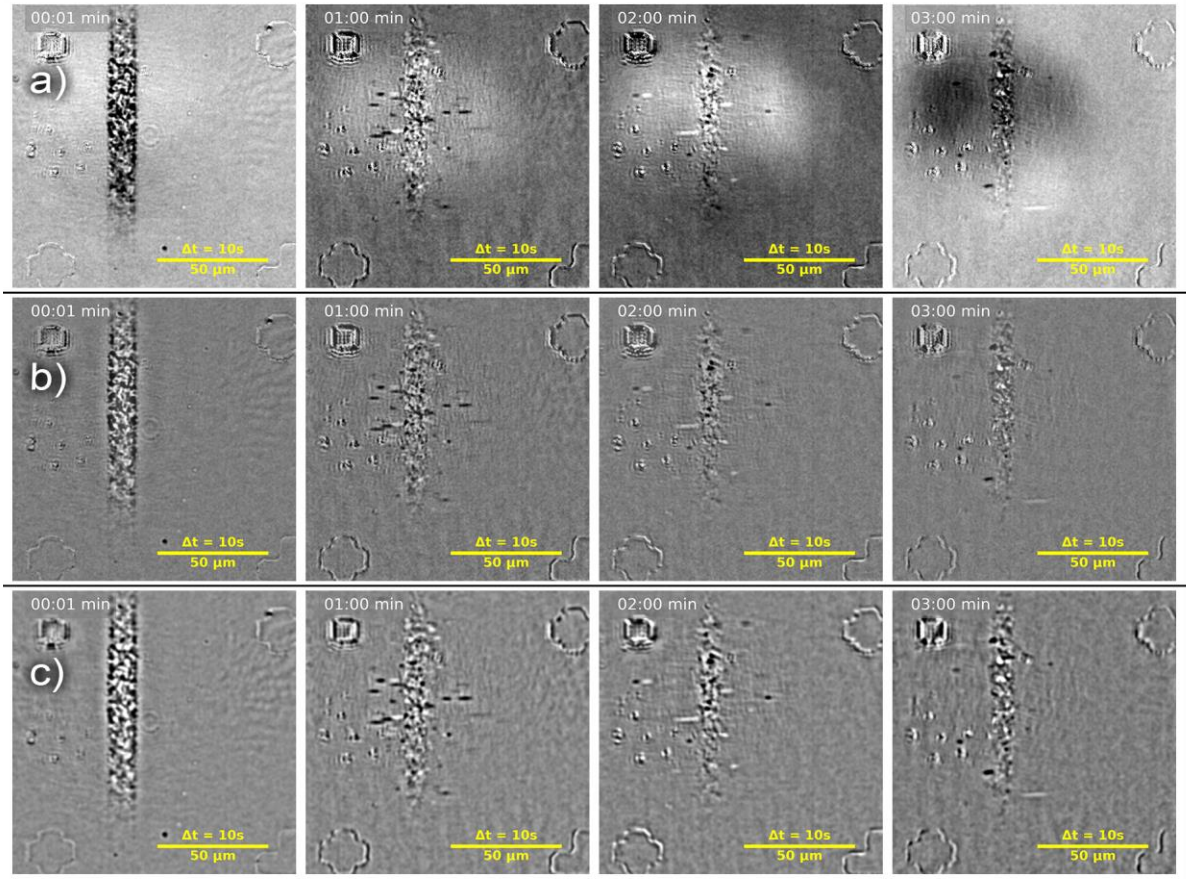

Figure S1: Snapshots of the video at different steps of video treatment. (a) After shade correction using differential treatment with 10 s time delay, (b) after FFT band-pass filtration, (c) after Gaussian difference edge enhancement.

The second and most crucial step of data treatment is shade correction, which compensates for the uneven illumination in the frame causing shading and vignetting [2]. The intensity distribution $I(x, y)$ of a given image can be described by the following equation:

$$I(x, y) = I^{\text{true}}(x, y) \cdot S(x, y) + D(x, y) \qquad \text{(S2)}$$

where x and y are the pixel coordinates, I^{true} is the change in intensity caused by nanotube absorption, S is a multiplicative term describing the uneven illumination of the image (flat field), and D is an additive term describing the image errors introduced by the camera itself (dark field) [3]. The impact of the latter term is negligible in our case. The average intensity of thermal noise from the camera in the absence of signal (i.e., measured with the lens cap on) is approximately 5% of the average signal intensity during experiments. Moreover, its contribution is uniform across the entire area of the camera sensor; hence, its effect can be neglected. The multiplicative term S in Equation S2, manifesting itself as dark vignette in the image area (see Figure S1a), is the major source of distortion on the raw videos. Its contribution can cover up to 80% of image color depth masking the contrast of tracked objects. Since the size of the nanotubes in the videos is much smaller than the uneven illumination, and their contrast in the raw videos is always lower than the background (see Figure S1a), the most appropriate method of image reconstruction is morphological multiplicative filtering [4]. In this case, the flat field can be described as follows [5]:

$$S(x, y) = I_{\text{ref}}(x, y) \cdot C_M \qquad \text{(S3)}$$

where $I_{\text{ref}}(x, y)$ is the morphological filter or reference frame for a processed image, and $C_M = \frac{1}{\mu\left(I_{ref}(x,y)\right)}$ is the normalization coefficient needed to restore the grayscale, which for our videos is inversely proportional to the mean intensity of the reference frame.

Thus, we used the following shade correction equation to restore the image:

$$I^{\mathrm{true}}(x,y) = I(x,y) \cdot \left(\frac{\mu\left(I_{\mathrm{ref}}(x,y)\right)}{I_{\mathrm{ref}}(x,y)}\right). \quad \text{(S4)}$$

An important aspect that affects the quality of the image is the choice of the reference frame. The standard method of shade correction (illustrated in Figure 1b) uses the same reference frame for the entire image sequence, most commonly, the first frame of the video before starting nanotube growth, which contains information about the initial unevenness of illumination. However, temporal and spatial drifts of the sample during in situ recording can cause a slow evolution of $S(x,y)$ over the experiment duration, via focus or absorption changes. This means that, with increasing time, the initial fixed frame becomes less appropriate for shade correction and that the contrast decreases. To overcome this problem, we developed a differential method of video treatment. In this method, the frame recorded at a fixed delay (typically from 5 to 30 s) before the treated frame is used as the reference. As shown in Figure 1c, such a differential or rolling-frame approach leads to better background subtraction and increased contrast. However, the difference between the reference and processed frames can lead to background heterogeneity due to light fluctuations on a time scale shorter than the delay time (Figure S1a).

To compensate for this residual unevenness of illumination as well as to clean “salt-n-pepper” noise, we used Fourier filtration [6,7]. For our purpose, the best parameter for fast Fourier transformation (FFT) band-pass filter was to preserve only the modes with sizes between 3 and 40 pixels. After reverse transformation, the images were free of both residual unevenness of illumination and fine noise (Figure S1b).

Nanotubes in Figure S2b (black and white horizontal segments) still lack sufficient sharpness. To enhance the contrast of the nanotube edges, we used a Gaussian band-

pass filter (difference of Gaussians) [8]. The best performance (see Figure S1c) was achieved with standard deviations of $\sigma_{\mathrm{min}} = 1{,}5$ and $\sigma_{\mathrm{max}} = 5$, found empirically.

The above-described method applied to fixed field-of-view videos allows for detecting changes occurring in the nanotubes during their growth (Figure S1c). In particular, a dark contrast corresponds to a local increase in light absorption (e.g., caused by nanotube elongation) while a bright contrast corresponds to a local decrease in absorption (e.g., caused by nanotube shrinkage or change of chirality toward a nanotube with a lower absorption cross section over the experimental spectral range). Since this method highlights only the changes occurring during the delay time, it clearly resolves in time nanotubes growing at the same location using the most appropriate delay time (Figure S2). Additionally, the length of the segment is proportional to the instantaneous growth rate of the nanotube and can be expressed using the following equation:

$$v = \frac{L_{\mathrm{seg}}}{\Delta t} \qquad \text{(S5)}$$

where L_{seg} is the segment length in micrometers, and Δt is the delay time. Another advantage of the differential treatment is the increase in the contrast of the nanotubes captured on the videos by almost an order of magnitude, as shown in Figure S3.

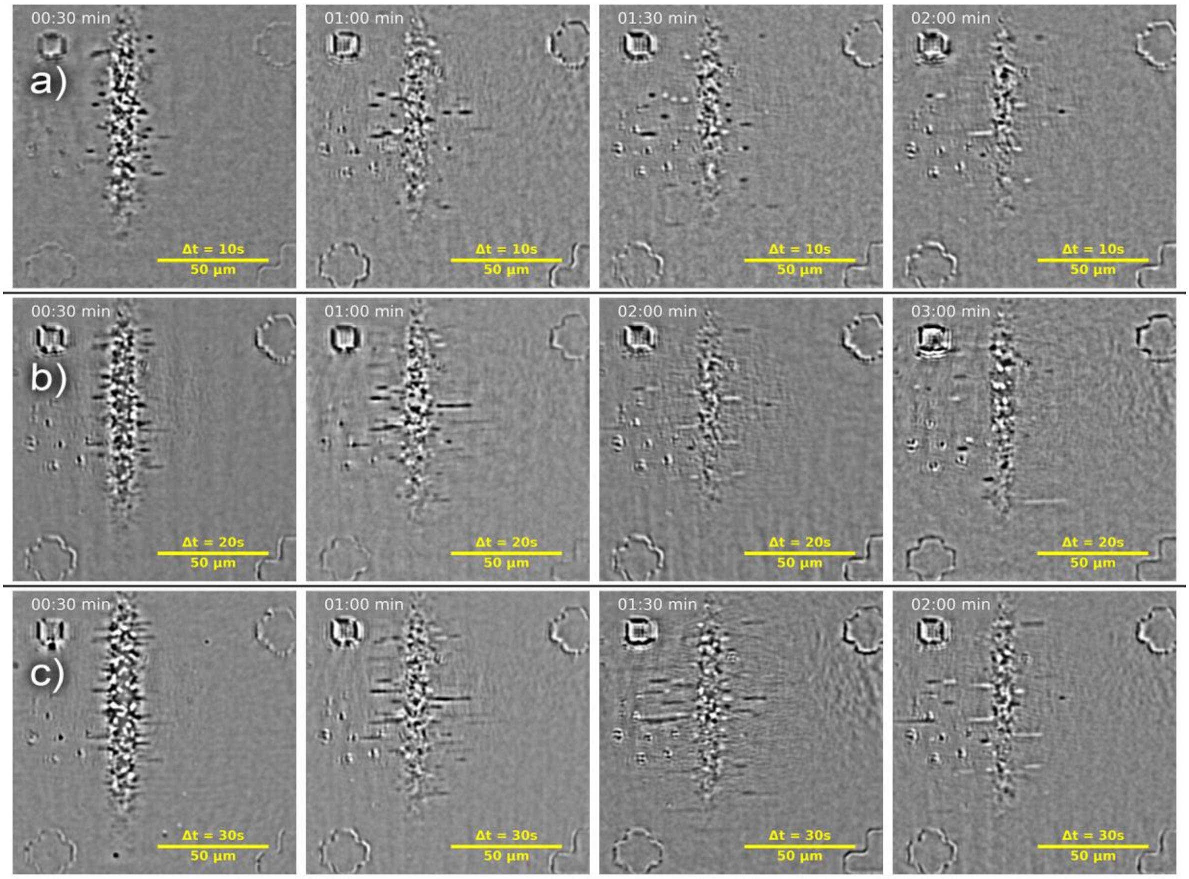

Figure S2: Several snapshots of a fully processed video with differential shade corrections with delay times of (a) 10 s, (b) 20 s, and (c) 30 s.

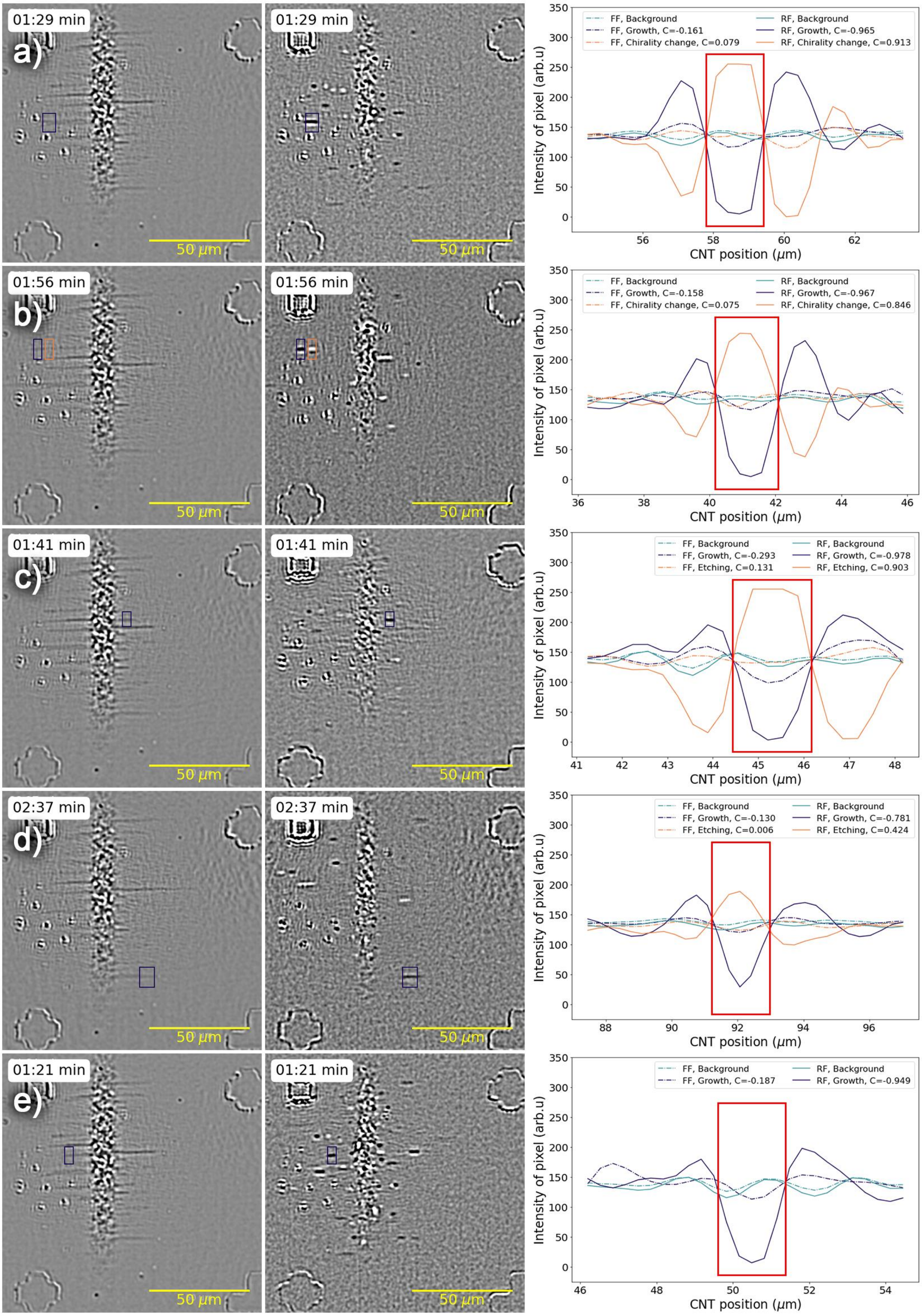

Figure S3: Snapshots of nanotube growth from videos with fixed-frame (FF) (left column) and rolling-frame (RF) processing (middle column). The plots in the right

column display the pixel intensity profiles averaged along the *x*-axis of the boxes shown in the left and middle columns. The red rectangle marks the nanotube's location. Purple lines represent the contrast of the growing nanotubes measured at the moments shown in the snapshots. Orange lines indicate chirality changes (rows a and b) or etching processes (rows c and d). The contrasts were calculated using snapshots taken when structural changes became visible. Delay times for each row are: (a) 42 s, (b) 0 s (orange box in images), (c) 12 s, and (d) 18 s. The background value (blue line) was obtained from the same region of interest in the frame at the fifth second of the video, before any nanotube growth occurred in the area.

Training process

The core of the system responsible for nanotube recognition is Mask-RCNN [9]. This AI architecture includes several neural networks working in the same flow, performing three tasks. Rescaling is done by a sequence of convolution and deconvolution layers of neural networks, or the backbone network. This part enables efficient detection of objects of different sizes. The results of its calculations feed into the region proposal network (RPN), which selects regions on the image where objects are most likely located. Then, the selected regions of interest (ROIs), together with data from the backbone, are fed to the input of fully connected convolutional network. As output, the model provides a mask of the object with the size of the original image, the bounding box, as well as the class of the object and the estimated reliability of the classification. The backbone in this model, can be chosen according to the required detection quality or the computational device's performance. We used the combination of ResNet-50 neural network with feature pyramid network [10,11], which provides high detection quality with moderate computation costs. To reduce training time [12], we used a backbone model pre-trained on the Microsoft COCO-2017 dataset [13].

The dataset contained 580 manually marked images and was split into two subsets: training (550 images) and validation (30 images). This number of images is sufficient for training a functional model. In biomedical image segmentation, it has been shown that training datasets as small as 30 images can yield robust recognition performance. This efficiency is largely attributed to the nature of pixel-wise mask prediction, where each pixel serves as a distinct training instance; thus, a single 1024 × 1024 image contributes over one million training points to the model [14,15]. In contrast, in situ homodyne microscopy images used in our study have lower resolution (approximately 400 × 400 pixels), and consequently provide fewer training entries per image. Based on this, we hypothesized that reliable segmentation performance in our case would require a minimum of ~50 annotated images.

To empirically evaluate this, we trained models on subsets of increasing size (5, 10, 25, 50, 100, 200, and 400 images). We observed that training on fewer than 100 images often led to rapid overfitting, characterized by quickly diminishing training loss coupled with poor generalization on the test set (see below in Figure S6 and Figure S7). As the training set increased, model performance improved, with the configuration trained on 400 images achieving results comparable to our full model trained on all 530 labeled images. Given this trend, where performance improves noticeably with approximately threefold increases in dataset size, we anticipate that the next significant performance gain would occur when training with approximately 1000 annotated images.

During the training of the model we also applied augmentations to increase the stability of recognition. For in situ videos, the most relevant transformations are horizontal and vertical flips, as well as changes in contrast and brightness within the limits observed in the videos (Figure S4). The probability of occurrence of each augmentation was 0.5, and each image could be transformed by several augmentations. The presence of

horizontal and vertical flips enhanced the ability of the model to localize the objects of interest, which is crucial since the positions of the catalyst line, optic marks, and carbon nanotubes vary from one synthesis to another. Brightness and contrast adjustments made the recognition process more robust regarding the quality of the videos (see the evaluation of the model trained on the augmented and non-augmented dataset in Table S1). Moreover, using these augmentations allows the neural network to recognize a larger number of variations of the input data without the necessity of manually marking down more images.

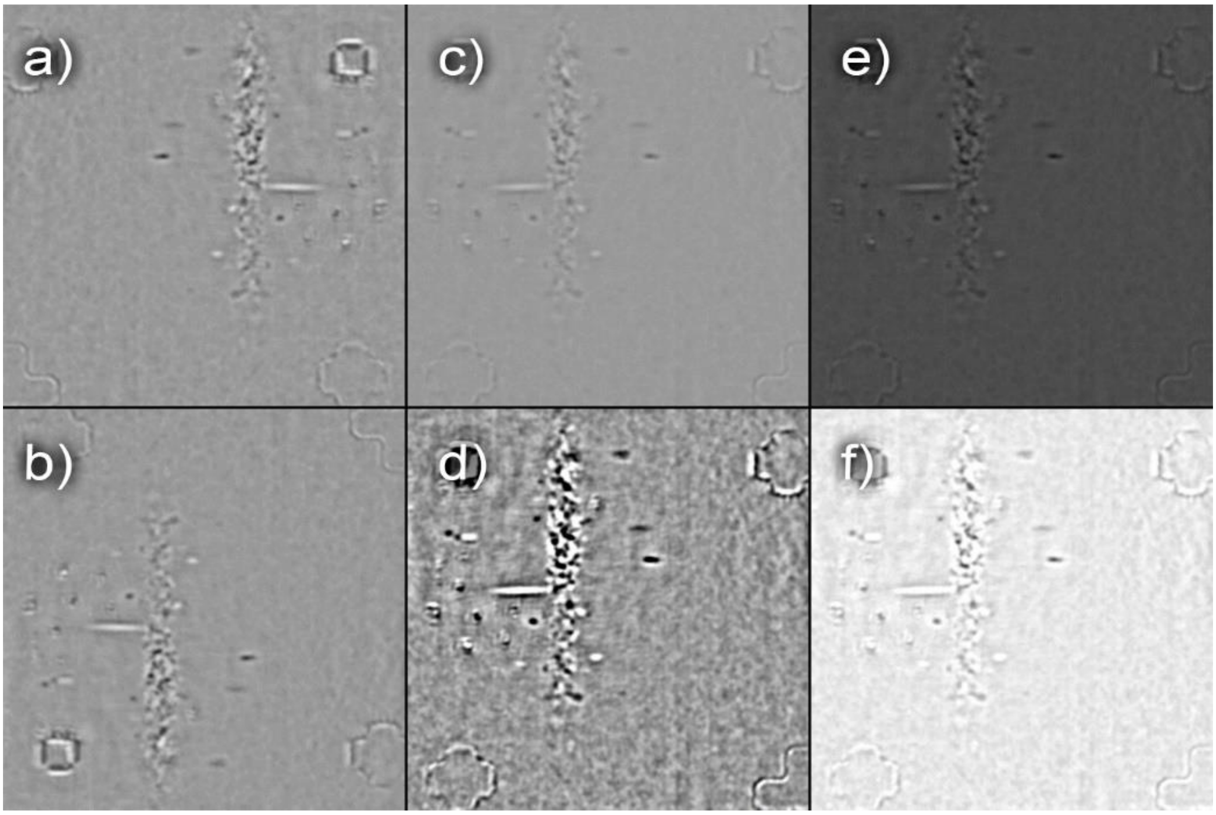

Figure S4: Video snapshots after various transformations used as augmentations of training dataset. (a) Horizontal and (b) vertical flips, adjustment of the contrasts between (c) minimum and (d) maximum, and brightness adjustment between (e) minimum to (f) maximum.

Table S1: Evaluation using mAP metric of the pretrained model trained for 150 epochs on the augmented dataset of 530 images (PTA) with the model trained from scratch on the same dataset with no augmentations applied (TFS) for the same number of epochs. Also included are performance metrics for models trained for 150 epochs on reduced training subsets of 10, 50, 100, 200, and 400 images. The numeric subscripts beside the metrics correspond to the intersection over union (IoU) values for recognized objects, and the alphabetic ones correspond to the sizes of recognized objects in pixels: S for small ($\text{area} < 32^2$), M for medium ($32^2 < \text{area} < 96^2$) and L for large ($\text{area} > 96^2$). AP_{COCO} was calculated for IoU in the range from 0.5 to 0.99 with a step of 0.05.

Parameter \ Metric		AP_{COCO}	AP_{50}	AP_{75}	AP_S	AP_M	AP_L
PTA	Boundary Box	50.1	83.3	53.3	37.9	46.3	56.0
	Mask	47.1	81.7	46.9	34.1	38.4	55.0
TFS	Boundary Box	47.1	79.6	48.2	36.3	39.8	50.7
	Mask	43.1	79.3	37.5	33.3	39.0	47.5
10	Boundary Box	32.3	56.4	32.6	13.1	26.8	49
	Mask	31.3	59	28.6	12.1	25.8	37.6
50	Boundary Box	40.0	70.1	39.8	29.4	30.6	41.5
	Mask	38.7	70.9	36.0	26.7	30.7	40.8
100	Boundary Box	47.8	80.3	47.3	36.6	48.3	48.2
	Mask	44.2	80.3	40.4	30.1	35.4	47.2
200	Boundary Box	47.7	81.0	47.5	35.0	37.4	49.1
	Mask	46.1	81.5	44.5	32.6	33.3	49.8
400	Boundary Box	49.1	82.2	52.3	37.2	42.7	51.6
	Mask	46.5	81.9	45.7	33.9	37.5	49.6

The speed of the model is about 3.5 frames per second while running on a GPU (NVIDIA GeForce GTX 1050), which makes it unsuitable for video streaming recognition. However, for our purposes, the recognition quality was the main goal. It meets the modern standards of NN for object detection as seen from benchmarks [16]. The resulting model detected segments corresponding to nanotube growth and changes in their structure, as well as optic marks and catalyst lines (Figure 2 and Figure S5). This process proceeds frame by frame through the video. Hence, to extract the kinetics of nanotube growth and structural changes, we applied a combination of the Hungarian method [17] and Kalman filter (or linear quadratic estimation) [18], widely used for object tracking [19]. The former was used to track masks on consecutive frames. However, some frames in the videos are unrecognizable due to imaging artifacts, illumination instability, or uncompensated vibrations. Therefore, after completing the first stage of tracking, all segments were grouped into clusters of sizes ranging from one to several tens of masks corresponding to consecutive frames, and a Kalman filter was applied to merge the groups of segments that correspond to the same nanotube.

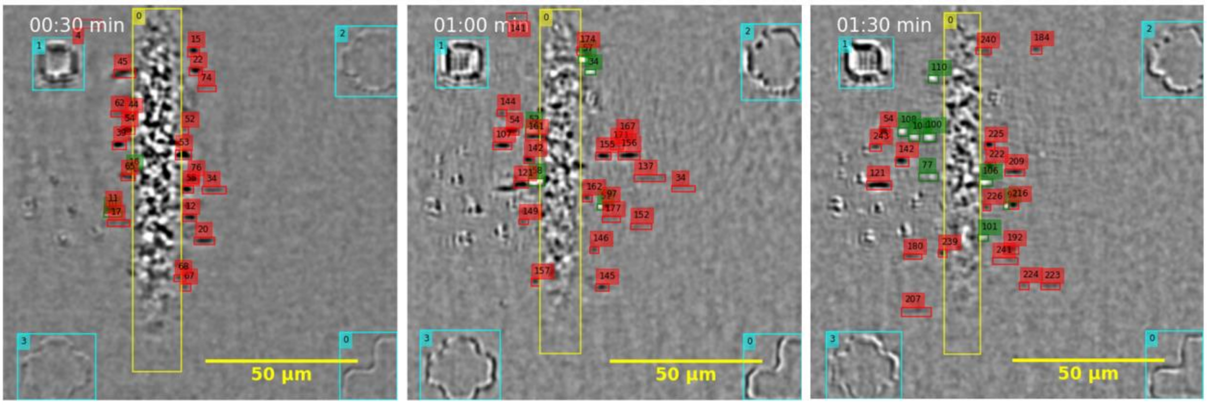

Figure S5: Snapshots from the in situ video with boundary boxes around recognized objects and their numbers in the dataset. Colors highlight growing nanotube segments (red), structural changes (green), catalyst line (yellow), and optical marks (blue).

Each group of segments tracked by the Hungarian method containing two or more masks was used to estimate the possible later or earlier location of other groups of masks corresponding to the same nanotube. These potential positions were calculated using the average length of all masks (l_i) and the locations of the first (for tracking back in time) and the last (for tracking forward in time) mask ($X(t_i)$). The growth rate ($v(t_i)$) was calculated as a ratio of the average length of all masks to the delay time of the current video, and the growth direction was determined by the sign of the coordinate differences of the beginning and end of the segment of the first and last mask in the group. If the signs were negative, such a group was excluded from consideration as a recognition artifact since every segment should move in the same direction. The width of the searched segment was defined as the maximum width of merged masks of the whole group.

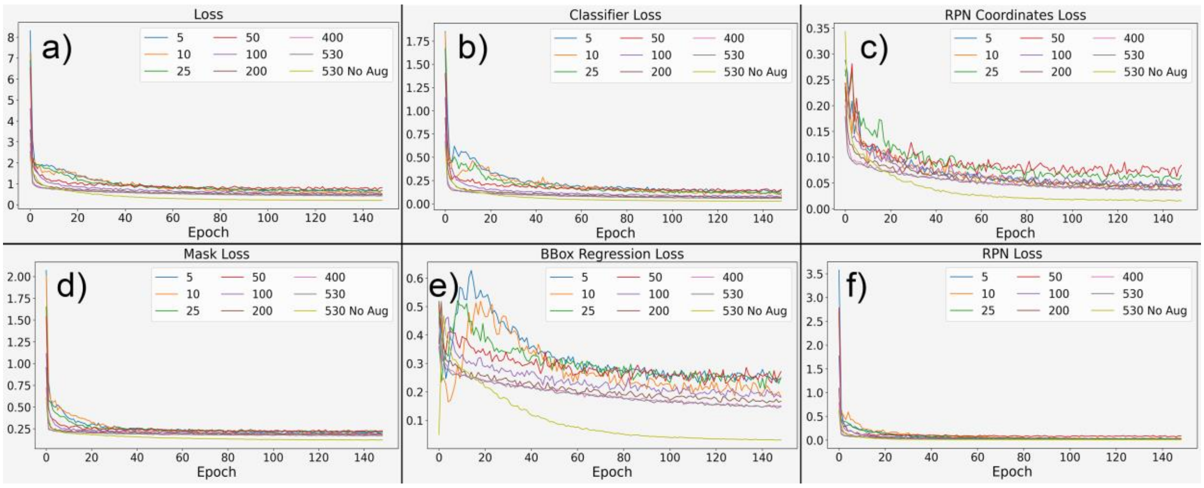

Figure S6: Training curves of models trained on different image sets (numbers in the legend) with augmentations and one instance of full model trained on the dataset without augmentations and without pretraining (530 No Aug). The images show the loss functions of (a) overall sum of all loss functions optimized during training, (b) RoI classifier, (c) region proposal network coordinates predictor, (d) mask predictor, (e) bounding box coordinates predictor, and (f) RPN object-background classifier.

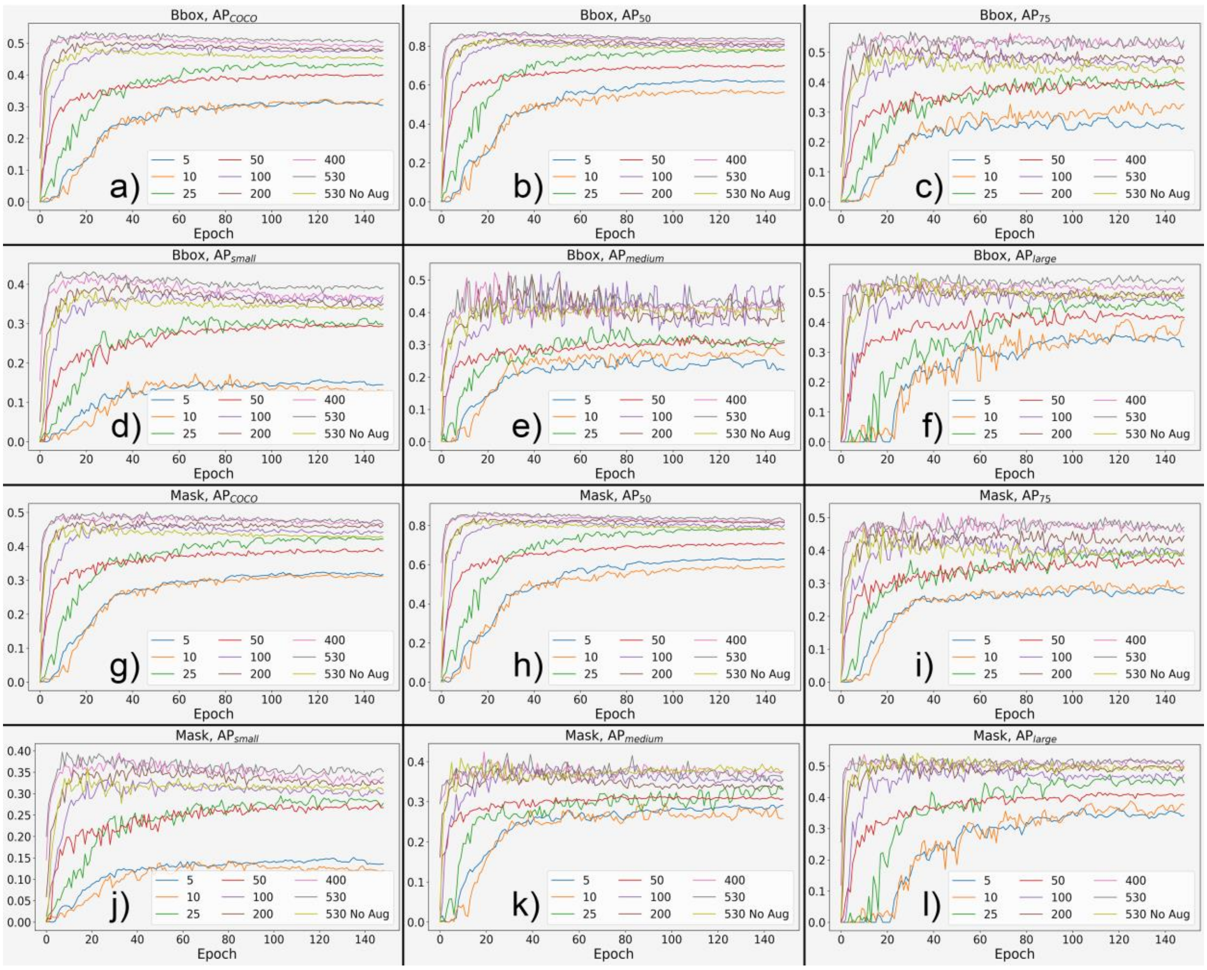

Figure S7: Test set evaluation curves for the models trained on subsets of different sizes (number of images shown in the legend) with augmentations, and one instance of full model trained on the dataset without augmentations and without pretraining (530 No Aug). The curves show the evolutions of the metrics demonstrated in Table S1.

References

1. Open Source Computer Vision. Template Matching. https://docs.opencv.org/4.5.2/d4/dc6/tutorial_py_template_matching.html (accessed November 2, 2024).

2. Goldman, D. B. *IEEE Trans. Pattern Anal. Mach. Intell.* **2010,** *32,* 2276–2288. doi:10.1109/TPAMI.2010.55
3. Peng, T.; Thorn, K.; Schroeder, T.; Wang, L.; Theis, F. J.; Marr, C.; Navab, N. *Nat. Commun.* **2017,** *8,* 14836. doi:10.1038/ncomms14836
4. Russ, J. C. *The Image Processing Handbook*, 5th ed.; CRC Press, 2006.
5. Tomazevic, D.; Likar, B.; Pernus, F. *J. Microsc. (Oxford, U. K.)* **2002,** *208,* 212–223. doi:10.1046/j.1365-2818.2002.01079.x
6. Brigham, E. O. *The fast Fourier transform and its applications;* Prentice Hall: Englewood Cliffs, NJ, USA, 1988.
7. Cooley, J. W.; Lewis, P. A. W.; Welch, P. D. *IEEE Trans. Educ.* **1969,** *12,* 27–34. doi:10.1109/TE.1969.4320436
8. Wang, B.; Rose, D. M.; Farag, A. A.; Delp, E. J. Local estimation of Gaussian-based edge enhancement filters using Fourier analysis. In *1993 IEEE International Conference on Acoustics, Speech, and Signal Processing;* April 27–30, 1993, Minneapolis Convention Center, Minneapolis, Minnesota, USA, Institute of Electrical and Electronics Engineers: New York, NY, Piscataway, NJ, USA, 1993.
9. He, K.; Gkioxari, G.; Dollár, P.; Girshick, R. Mask R-CNN. In *2017 IEEE International Conference on Computer Vision (ICCV);* 2017; pp 2980–2988. doi:10.1109/ICCV.2017.322
10. He, K.; Zhang, X.; Ren, S.; Sun, J. Deep Residual Learning for Image Recognition. In *2016 IEEE Conference on Computer Vision and Pattern Recognition (CVPR)*, 2016; pp 770–778. doi:10.1109/CVPR.2016.90
11. Lin, T.-Y.; Dollár, P.; Girshick, R.; He, K.; Hariharan, B.; Belongie, S. Feature Pyramid Networks for Object Detection. In *2017 IEEE Conference on*

Computer Vision and Pattern Recognition (CVPR); 2017; pp 936–944. doi:10.1109/CVPR.2017.106

12. George Karimpanal, T.; Bouffanais, R. *Adaptive Behavior* **2019,** *27*, 111–126. doi:10.1177/1059712318818568
13. Lin, T.-Y.; Maire, M.; Belongie, S.; Hays, J.; Perona, P.; Ramanan, D.; Dollár, P.; Zitnick, C. L. Microsoft COCO: Common Objects in Context. In *Computer Vision – ECCV 2014;* pp 740–755. doi:10.1007/978-3-319-10602-1_48
14. Ronneberger, O.; Fischer, P.; Brox, T. U-Net: Convolutional Networks for Biomedical Image Segmentation. In *Medical Image Computing and Computer-Assisted Intervention – MICCAI 2015;* pp 234–241. doi:10.1007/978-3-319-24574-4_28
15. Ghosh, P.; Mitchell, M. Segmentation of medical images using a genetic algorithm. In *Proceedings of the 8th annual conference on Genetic and evolutionary computation;* 2006; pp 1171–1178. doi:10.1145/1143997.1144183
16. Papers With Code. Benchmark of neural network for object detection. https://paperswithcode.com/sota/real-time-object-detection-on-coco (accessed November 2, 2024).
17. Kuhn, H. W. *Nav. Res. Logist. Q.* **1955,** *2,* 83–97. doi:10.1002/nav.3800020109
18. Kalman, R. E. *J. Basic Eng.* **1960,** *82,* 35–45. doi:10.1115/1.3662552
19. Bewley, A.; Ge, Z.; Ott, L.; Ramos, F.; Upcroft, B. Simple online and realtime tracking. In *2016 IEEE International Conference on Image Processing (ICIP);* 2016; pp 3464–3468. doi:10.1109/ICIP.2016.7533003
